# Efficacy of neuromuscular training for enhancing postural stability in young athletes: a systematic review and meta-analysis

**DOI:** 10.3389/fphys.2026.1827904

**Published:** 2026-05-21

**Authors:** Junfeng Zhou, Shuaichao Liu, Han Sun, Xiaohong Zheng

**Affiliations:** Institute of Physical Education and Training, Capital University of Physical Education and Sports, Beijing, China

**Keywords:** injury prevention, meta-analysis, motor control, neuromuscular training, postural stability, young athletes

## Abstract

**Background:**

Postural stability is vital for athletic performance and injury prevention in young athletes. While neuromuscular training (NMT) is common, its specific effects on different stability components and optimal training parameters remain unclear. This systematic review and meta-analysis evaluated the efficacy of NMT on dynamic and static postural stability in young athletes.

**Methods:**

Five databases (PubMed, Web of Science, Embase, Cochrane Library, and Scopus) were searched for randomized controlled trials (RCTs) examining NMT effects on postural stability in young athletes. Methodological quality and risk of bias were assessed using the PEDro scale and RoB 1.0. Evidence certainty was evaluated via the GRADE approach. Data were pooled using a random-effects model, reporting standardized mean differences (SMDs) and 95% confidence intervals (CIs).

**Results:**

Eighteen articles (19 independent trials, *N* = 605) were included. NMT significantly improved both dynamic [SMD = 0.96, 95% CI (0.70, 1.22), *p* < 0.00001] and static postural stability [SMD = 0.96, 95% CI (0.60, 1.32), *p* < 0.00001]. Subgroup analyses identified participant age as a significant source of heterogeneity for static stability outcomes.

**Conclusions:**

NMT effectively enhances dynamic and static postural stability in young athletes. Given the comparable efficacy across different NMT modalities, practitioners can flexibly design training programs to suit specific athletic contexts and practical constraints.

**Systematic Review Registration:**

https://www.crd.york.ac.uk/PROSPERO/view/, identifier CRD420261299111.

## 1 Introduction

Postural stability is essential for executing complex motor tasks and preventing musculoskeletal injuries (Hewett et al., 2005; Horak, 2006; Paillard, 2019). However, young athletes face unique challenges during the physiological process of maturation. The adolescent growth spurt is characterized by rapid, asynchronous increases in limb length and body mass (Malina et al., 2004), often precipitating a “neuromuscular lag.” During this phase, the maturation of motor control and proprioceptive systems fails to keep pace with accelerated skeletal growth (Quatman et al., 2006; Parsons, 2014; Parry et al., 2024). Biomechanically, limb elongation increases the segmental moment of inertia, while an elevated center of mass compromises stability—a phenomenon termed “adolescent awkwardness” (McKay et al., 2016; Borato et al., 2025). During this window, athletes may experience a transient regression in sensorimotor function and diminished joint position acuity (Williams et al., 2021). Consequently, altered biomechanics during high-risk maneuvers, such as jumping or cutting, can amplify knee valgus moments and heighten the risk of anterior cruciate ligament (ACL) tears and ankle sprains (Hewett et al., 2010; Wordeman, 2014; Gu et al., 2025).

Neuromuscular training (NMT) optimizes motor command output by stimulating sensory pathways and inducing central nervous system adaptations (Faigenbaum et al., 2011; Yang et al., 2025). Contemporary Integrative Neuromuscular Training (INT) models have moved beyond single-modality exercises to combine core stability, plyometrics, balance, and agility drills (Myer et al., 2011; Faude et al., 2017). These programs enhance postural stability through “sensory reweighting” (Peterka, 2002). By introducing destabilizing stimuli, such as unstable surfaces or visual occlusion, NMT challenges the nervous system, promoting an increased reliance on proprioceptive and vestibular inputs. This structured motor training promotes neuroplasticity, optimizing functional connectivity within the frontoparietal network that governs motor learning (Vacchini et al., 2025). Given the heightened neural plasticity of adolescence, NMT provides a critical stimulus for developing robust postural control circuitry.

While previous meta-analyses establish NMT’s efficacy in reducing lower extremity injuries by approximately 36% and ACL injuries by nearly 50% (Emery et al., 2015), evidence regarding its specific impact on postural stability remains heterogeneous. This inconsistency largely stems from variations in NMT modalities, intervention dosages, and outcome measures (Behm et al., 2015; Gebel et al., 2018). Furthermore, dynamic postural stability (e.g., Star Excursion Balance Test, Y-Balance Test) and static postural stability (e.g., Balance Error Scoring System, stabilometry) may respond differently to specific NMT components (Imai et al., 2014; Pinzón-Romero et al., 2019; Zhang et al., 2021; Daneshjoo et al., 2022; Gasim et al., 2022). Therefore, this systematic review and meta-analysis aimed to quantify the effects of NMT on dynamic and static postural stability in young athletes compared to conventional training. A secondary objective was to explore the moderating effects of NMT modality, intervention duration, outcome measures, and participant age, ultimately providing a robust empirical rationale for optimizing youth athletic development programs.

## 2 Protocol and registration

This systematic review and meta-analysis followed the Preferred Reporting Items for Systematic Reviews and Meta-Analyses (PRISMA) guidelines (Page et al., 2021). The study protocol was prospectively registered in PROSPERO (CRD420261299111). All methodological procedures were established *a priori* to maintain transparency and minimize bias.

## 3 Data sources and search strategy

Two independent reviewers (JFZ and SCL) searched PubMed, Web of Science, Embase, the Cochrane Library, and Scopus from inception to November 1, 2025. The search strategy combined Medical Subject Headings (MeSH) and free-text terms using Boolean operators (AND/OR) (the full search strategy is provided in the **Supplementary Files**). Keywords targeted the population (e.g., “adolescent”, “young athletes”), interventions (e.g., “neuromuscular training”, “integrative neuromuscular training”), and outcomes (e.g., “postural stability”, “Y-balance test”). Reference lists of the retrieved articles were manually screened to identify additional eligible trials.

**Figure 1:**
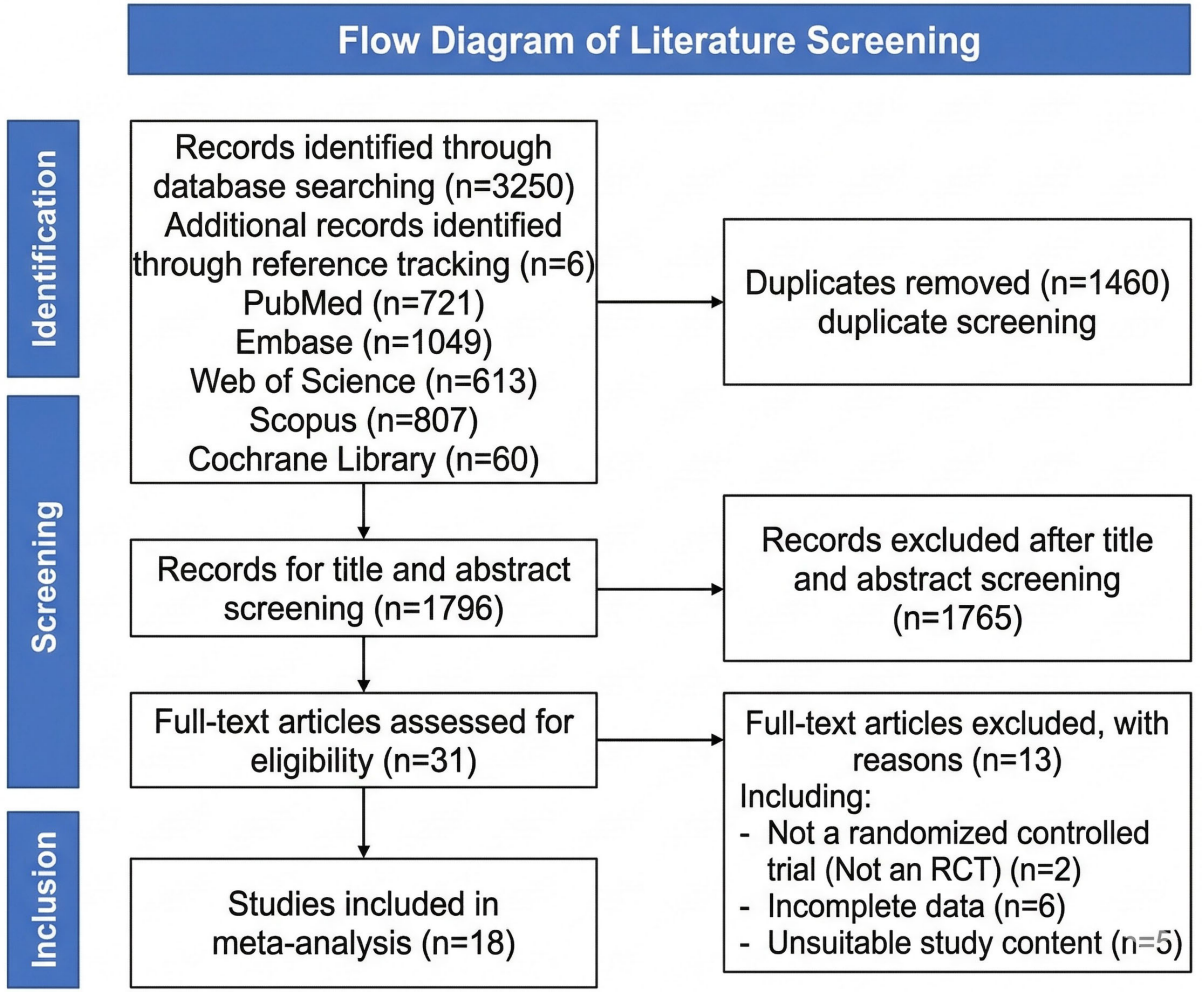
PRISMA flow diagram of the study selection process.

The initial search yielded 3256 records. After removing 1460 duplicates via NoteExpress 3.2.0, 1765 titles and abstracts were screened, leaving 31 articles for further evaluation. Following a full-text assessment, 13 articles were excluded, resulting in 18 randomized controlled trials (RCTs) for the quantitative synthesis (**Figure 1**).

## 4 Inclusion and exclusion criteria

Eligibility criteria were defined using the PICOS framework (Chandler et al., 2019):

Population (P): Young athletes (10–24 years; UN/WHO definition), encompassing the developmental continuum from early adolescence to late-stage neuromuscular consolidation in collegiate participants.

Intervention (I): Multi-component NMT programs emphasizing neuromuscular coordination, irrespective of specific training duration, frequency, or modality.

Comparison (C): Conventional sport-specific training or other active/passive regimens lacking NMT components (e.g., traditional strength training). The NMT intervention could either supplement or substitute a segment of the routine training.

Outcomes (O): Quantitative measures of dynamic postural stability (e.g., Star Excursion Balance Test, Y-Balance Test, instrumented platforms) or static postural stability (e.g., Balance Error Scoring System, stabilometry, single-leg stance tests).

Study Design (S): Randomized controlled trials (RCTs) only.

Exclusion criteria comprised (1): non-RCT designs or review articles (2); animal models (3); unavailable full texts; and (4) insufficient or non-extractable data for quantitative synthesis.

## 5 Data extraction and processing

Two independent reviewers (JFZ and SCL) extracted data into a standardized spreadsheet, capturing participant characteristics, intervention details, and pre- and post-intervention outcomes (means, standard deviations [SDs], and mean change scores). Study characteristics are summarized in **Tables 1**, **2**. Discrepancies were resolved through discussion or consultation with a third senior researcher.

**Table 1:** Participant characteristics and outcome measures of the included studies.

Study	*N*	Age (EG/CG), years	Sex	Sport	Outcomes
Esmailnezhad et al. (2024)	24	15.5 ± 0.9/15.91 ± 0.79	F	Wrestling	YBT, BESS
Gong et al. (2024)	30	15.8 ± 0.79/15.6 ± 0.7	M	Basketball	SEBT, SLST-EC
Kim et al. (2024)	30	16-19	M	Baseball	YBT
Mitrousis et al. (2023)	42	12.71 ± 0.41/12.73 ± 0.46	M	Soccer	Lafayette Platform, Johnson & Nelson Test
Shi et al. (2023)	16	13.7 ± 0.39/13.6 ± 0.33	M	Tennis	LOS
Sikora and Linek (2022)	90	12.5 ± 2.2/12.4 ± 2.1	M	Soccer	YBT, ALFA Platform
Aloui et al. (2022)	34	14.6 ± 0.5/14.6 ± 0.4	M	Soccer	YBT
Daneshjoo et al. (2022)	24	14.75 ± 1.1/14.58 ± 0.51	M	Handball	YBT, BBS, SLST
Gasim et al. (2022)	18	17.2 ± 0.4/17.3 ± 0.5/17.7 ± 0.5	M	Soccer	YBT
Gidu et al. (2022)	16	15.3 ± 3.0/13.6 ± 4.9	M	Soccer	BESS
Dogan and Savaş (2021)	30	12-14	M	Basketball	YBT, Stabilometer
Puzi and Choo (2021)	30	14.13 ± 0.83/13.6 ± 0.91	M/F	Handball	SEBT
Zhang et al. (2021)	58	19.81 ± 1.72/19.02 ± 1.97	M/F	Dance	YBT
Zacharakis et al. (2020)	25	13-14	M	Basketball	Lafayette Platform, Narrow Beam SLST
Pinzón-Romero et al. (2019)	58	12.93 ± 1.4/13.21 ± 1.3	M/F	Roller Skating	SEBT, BESS
Ondra et al. (2017)	21	17.3 ± 1.3/16.5 ± 1.8	M	Basketball	COP Velocity
Ramírez-Campillo et al. (2015)	40	11.2 ± 2.3/11.4 ± 2.4男	M	Soccer	COP Trajectory
Imai et al. (2014)	19	16.5 ± 0.5/16.1 ± 0.6	M	Soccer	SEBT, COP Trajectory

EG, experimental group; CG, control group; M, male; F, female; YBT, Y-Balance Test; SEBT, Star Excursion Balance Test; BESS, Balance Error Scoring System; LOS, Limits of Stability; SLST, Single-Leg Stance Test; SLST-EC, Single-Leg Stance Test with eyes closed; BBS, Berg Balance Scale; COP, Center of Pressure.

**Table 2:** Characteristics of the neuromuscular training interventions.

Study	Duration & Freq	Interventions (EG vs. CG)	Training content	Training protocol
Esmailnezhad et al. (2024)	8 wks, 3x/wk	EG (NMT): Core Stability [Primary], Balance & Proprioception [Primary], Dynamic Strength & Functional, Agility & Reactive Control CG: Routine warm-up	NMT: 3 segments (14 exercises). Pt 1: slow stretches/bridges; Pt 2: core/shoulder/leg/balance (3 difficulty levels); Pt 3: wrestling simulations. CG: Jogging, running, stretching.	NMT: Pre-training execution. Started at Level 1, progressed upon quality mastery. Emphasized posture control. CG: Time-matched routine warm-up.
Gong et al. (2024)	10 wks, 3x/wk	EG (NMT): Core Stability [Primary], Balance & Proprioception CG (TST): Traditional Strength Training	NMT: Isotonic/dynamic (4-point touch), static (suspension row), and dynamic unstable (Swiss ball push-ups). TST: Bodyweight (push-ups, planks) and resisted training (band sprints, weighted squats).	NMT & TST: 70–80% HRmax. NMT Prog: Wk 1 (15 reps × 3 sets, 30s rest); Wk 2 added 30s × 3 static holds; progressed to unstable surfaces. TST Prog: Added band resistance from Wk 4.
Kim et al. (2024)	6 wks, 3x/wk	EG(NMT): Plyometrics [Primary], Balance & Proprioception, Dynamic Strength & Functional CG: Kettlebell training	NMT: Combined plyometrics (split squat jumps, drop jumps) with kettlebell (KB) exercises (swings, snatches). CG: KB exercises only.	NMT: 40 min/session (20 min plyo + 20 min KB). Progressive intensity and complexity weekly. CG: 40 min single-mode training.
Mitrousis et al. (2023)	8 wks, 3x/wk	EG (NMT): Balance & Proprioception [Primary], Plyometrics, Dynamic Strength & Functional, Agility & Reactive Control CG (PT): Placebo training	NMT: Single-leg stance with multi-directional swings, flamingo balance, Bosu high knees, single-leg side hops, single-leg star hops. PT: Seated wall-ball catches, drop catches, rapid visual touch responses.	NMT: Post-routine training. 2 sets. Bi-weekly difficulty progression (e.g., eyes closed). Wk 1–2: 30s work/30s rest; Wk 3–8: 45s/45s. PT: 4 sets, 45s/45s.
Shi et al. (2023)	12 wks, 3x/wk	EG (NMT): Plyometrics [Primary], Balance & Proprioception, Agility & Reactive Control CG: Routine warm-up	NMT: Jump rope (forward/backward alternate, cross, single-leg squat jumps) + specific warm-up. CG: Specific warm-up (side shuffles, cross steps, backward curves, etc.).	Total 120 min/session. Pt 1 (30 min): 10 min jog/stretch + 20 min intervention (10 min rope + 10 min specific). Rope: 120s work/30s rest. CG: 10 min jog/stretch + 20 min specific warm-up.
Sikora and Linek (2022)	10 wks, 2x/wk	EG (NMT): Balance & Proprioception [Primary], Core Stability, Dynamic Strength & Functional CG: Routine soccer training	NMT: 5 stations: “Star” balance, Swiss ball limb raises, sensory disc rolls, Domyos board tilts, unstable platform squats with ball. CG: Routine club training.	NMT: Pre-training. 10 min bike warm-up + 45 min sensorimotor circuit. 4 sets × 8 reps, 10s rest. CG: Maintained routine without extra physical training.
Aloui et al. (2022)	8 wks, 2x/wk	EG (NMT): Plyometrics [Primary], Agility & Reactive Control, Dynamic Strength & Functional CG: Routine soccer training	NMT: 4-station circuit: 0.4m hurdle jumps + 15m sprint; 0.3m lateral jumps + 10m sprint; bounding + 15m sprint; single-leg hops + 10m sprint. CG: Technical/tactical + school PE.	NMT: 15–30 min circuit replacing technical-tactical segment. Total ground contacts progressed from 72 (Wk 1) to 144 (Wk 8). 90s rest between sets. CG: Routine in-season protocol.
Daneshjoo et al. (2022)	8 wks, 3x/wk	EG (NMT): Core Stability [Primary], Balance & Proprioception [Primary], Plyometrics, Agility & Reactive Control CG: Routine warm-up	NMT: 3 parts: Pt 1 (8 min): dynamic stretch/lunges/crawls; Pt 2 (10 min): 3 levels of V-sits, back extensions, single-leg balance, med-ball throws; Pt 3 (4 min): cutting/bounding. CG: Cross-field running, static stretch.	NMT: 20–25 min replacing standard warm-up. Progressive via 3 difficulty levels to accommodate varying baseline abilities. CG: Routine in-season warm-up.
Gasim et al. (2022)	8 wks, 2x/wk	EG1 (NMT): Core Stability EG2 (NMT): Plyometrics CG: Routine soccer training	NMT1: Planks, side planks, dead bugs, bird dogs, bridges. NMT2: Squat jumps, tuck jumps, lateral hops, box jumps, drop jumps. CG: Routine club training.	NMT1 & 2: 3 specific sessions/week post-standardized warm-up (15 min). CG: Maintained routine without extra core/plyo training.
Gidu et al. (2022)	8 wks, 4x/wk	EG (NMT): Balance & Proprioception [Primary], Dynamic Strength & Functional, Agility & Reactive Control	NMT: 2 sub-protocols. No-ball: Bosu squats, single-leg swings/hops, jumping lunges. With-ball: Bosu kicks/headers, resisted band kicks, dribbling around Bosu. CG: Technical/athletic training.	NMT: Executed on natural grass; manipulated surfaces (hard vs. foam). 4 sets × 10 reps (or 10/leg), 30s rest. CG: Time-matched routine training.
Dogan and Savaş (2021)	8 wks, 3x/wk	EG (NMT): Core Stability [Primary], Balance & Proprioception CG: Routine basketball training	NMT: Planks, bridges, jackknifes, bird dogs, side planks, band squats, med-ball twists, Bosu single-leg passes. CG: Routine specific skills training.	NMT: 45–60 min supplementary. 40–60% intensity. Wk 1–2: 3 sets/exercise. Added 1 set bi-weekly (6 sets by Wk 7–8). 20s work, 1:1 W/R ratio. CG: Routine training only.
Puzi and Choo (2021)	6 wks, 3x/wk	EG (NMT): Balance & Proprioception [Primary], Plyometrics [Primary], Agility & Reactive Control	NMT: Single-leg touches, jumps/catches; knee tucks, lateral bounding, supine bridges; 20m 4-station sprints, 20m zig-zags, resisted hip turns/shots. CG: Routine technical/tactical drills.	NMT: Progressive difficulty: from basic single-leg support (Wk 1) to reactive sprints and resisted specific skills (e.g., resisted cuts/shots). CG: Time-matched routine training.
Zhang et al. (2021)	10 wks, 3x/wk	EG (NMT): Balance & Proprioception [Primary], Core Stability [Primary], Dynamic Strength & Functional, Agility & Reactive Control CG: Routine dance warm-up	NMT: Dynamic warm-up (high knees, lateral shuffles); core/leg strength (planks, squats); balance (single-leg, unstable surfaces, eyes closed). CG: Dance-specific footwork and basic stretches.	NMT: 20 min replacing routine dance warm-up. Progressed by adding unstable surfaces or visual occlusion. CG: Pre-training routine warm-up.
Zacharakis et al. (2020)	8 wks, 3x/wk	EG (NMT): Balance & Proprioception [Primary], Plyometrics, Agility & Reactive Control CG: Routine basketball training	NMT: Single-leg balance/swings, flamingo, Bosu steps. Later added: eyes closed, simulated passing/shooting on balance board, trampoline hops. CG: Routine basketball training.	NMT: Post-training. 2 sets. Wk 1–2: 30s W/30s R, 2 min rest between sets; Wk 3–6: 45s W/45s R; Wk 7–8: 60s W/60s R. CG: Maintained routine without balance training.
Pinzón-Romero et al. (2019)	12 wks, 3x/wk	EG (NMT): Balance & Proprioception [Primary], Dynamic Strength & Functional, Agility & Reactive Control CG: Routine warm-up	NMT: 5 proprioceptive exercises (8 difficulty levels). Advanced stages included roller-skating balance with external perturbations (partner, balloons). CG: Jogging, multidirectional jumps, stretching.	NMT: 5-wk basic + 7-wk specific mesocycle. Progressed from stable to unstable surfaces to roller skates. Included progressive jump heights (5–15 cm). CG: Coach-prescribed pre-training warm-up.
Ondra et al. (2017)	20 wks, 3x/wk	EG (NMT): Balance & Proprioception [Primary], Core Stability, Plyometrics, Dynamic Strength & Functional CG: Routine warm-up	NMT: Warm-up: dynamic stretches. Array 1: iso squats + squat jumps, planks, iso lunges. Array 2 (circuit): Bosu iso squats, Swiss ball straight-arm planks, KB single-leg deadlifts. CG: Routine warm-up.	NMT: In-season. 1 session pre-training, 2 intra-training. Sequential execution: warm-up -> Array 1 -> Array 2 (2 rounds, 30s/exercise). CG: Load-matched routine training.
Ramírez-Campillo et al. (2015)	7 wks, 2x/wk	EG (NMT): Plyometrics [Primary], Balance & Proprioception, Dynamic Strength & Functional CG: Routine soccer training	NMT: Concurrent vertical and horizontal jumping exercises. CG: Routine soccer training.	NMT: Replaced 15–30 min technical/tactical training. Progressive loading. 30–120s rest between sets. CG: No plyometric or strength training permitted.
Imai et al. (2014)	12 wks, 3x/wk	EG (NMT): Core Stability [Primary], Balance & Proprioception CG (TST): Traditional Strength Training	NMT: Front planks, quadruped exercises, supine bridges, side planks. TST: Sit-ups (2 variations), back extensions (2 variations).	NMT: Post-training. Neutral spine focus. Wk 1–2: 60s × 2 sets. Progressed via single-limb elevation. CG: Time-matched. Wk 1–2: 40s × 3 sets max reps. Progressed by duration or posture.

EG, experimental group; CG, control group; NMT, neuromuscular training; TST, traditional strength training; PT, placebo training; KB, kettlebell; W/R, work/rest; Prog, progression; Iso, isometric.

Data transformations, including the aggregation of multiple correlated testing conditions (e.g., various stances, surfaces, or test variations) into composite means and SDs, and the imputation of missing change score SDs (assuming a conservative intra-individual correlation coefficient of r = 0.5), were conducted in accordance with the Cochrane Handbook for Systematic Reviews of Interventions (Higgins et al., 2019).

Specifically, to prevent disproportionate weighting from studies reporting multiple testing conditions, the following aggregation formulas were applied:


$${\bar{X}}_{comp}=\frac{1}{k}\sum_{i=1}^{k}{\bar{X}}_{i}$$

$$SD_{comp}=\sqrt{{\left(\frac{1}{k}\right)}^{2}(\sum_{i=1}^{k}SD_{i}^{2}+\sum_{i\neq j}r\cdot SD_{i}\cdot SD_{j})}$$

When pre-to-post intervention change scores were not reported, they were estimated as follows:


$${\text{Mean}}_{chg}={\text{Mean}}_{post}-{\text{Mean}}_{pre}$$

$$SD_{chg}=\sqrt{SD_{pre}^{2}+SD_{post}^{2}-(2\times r\times SD_{pre}\times SD_{post})}$$

## 6 Methodological quality assessment

Two independent reviewers (JFZ and SCL) evaluated study quality and risk of bias using the Physiotherapy Evidence Database (PEDro) scale (Maher et al., 2003) and the Cochrane Risk of Bias tool (RoB 1.0) (Higgins et al., 2011). Based on the 10-point PEDro scale, study quality was classified as excellent (9–10), high (6–8), moderate (4–5), or low (< 4). Concurrently, RoB 1.0 classified trials into Grade A (≥ 4 low-risk domains), Grade B 2–3), or Grade C (≤ 1).

**Figure 2:**
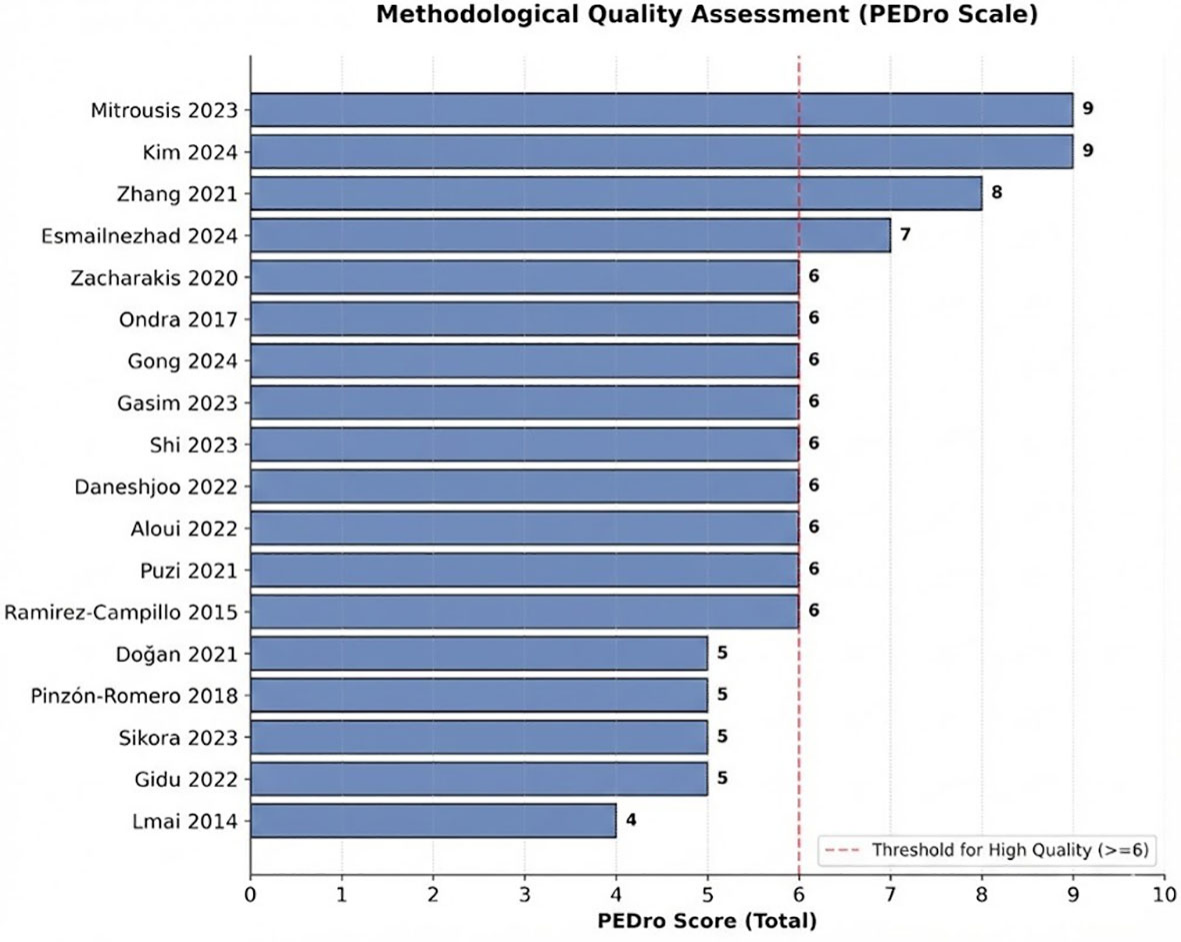
Methodological quality assessment of the included studies based on the PEDro scale.

**Figure 3:**
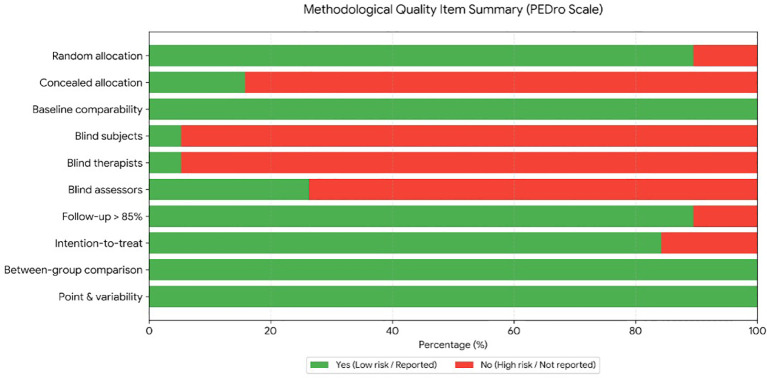
Summary of methodological quality criteria across the included studies (PEDro scale).

**Figure 4:**
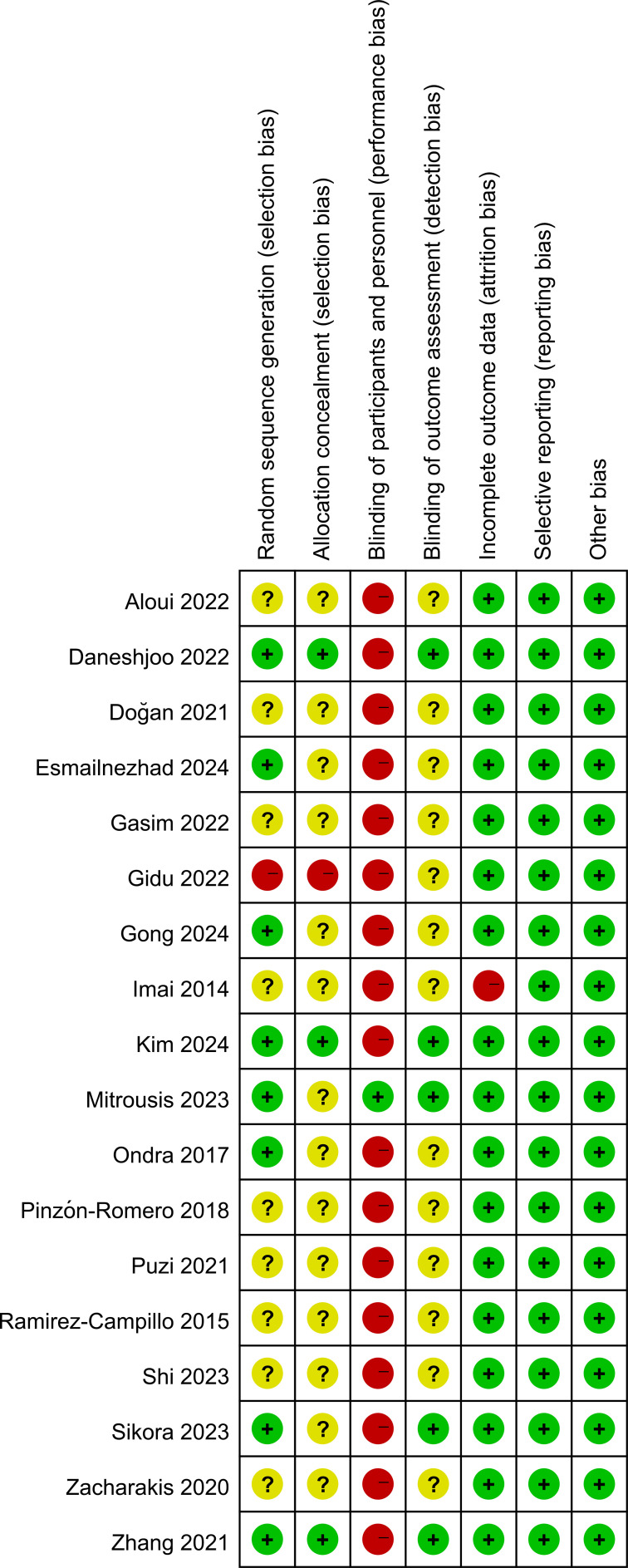
Risk of bias summary: review authors’ judgements about each risk of bias item for each included study.

**Figure 5:**
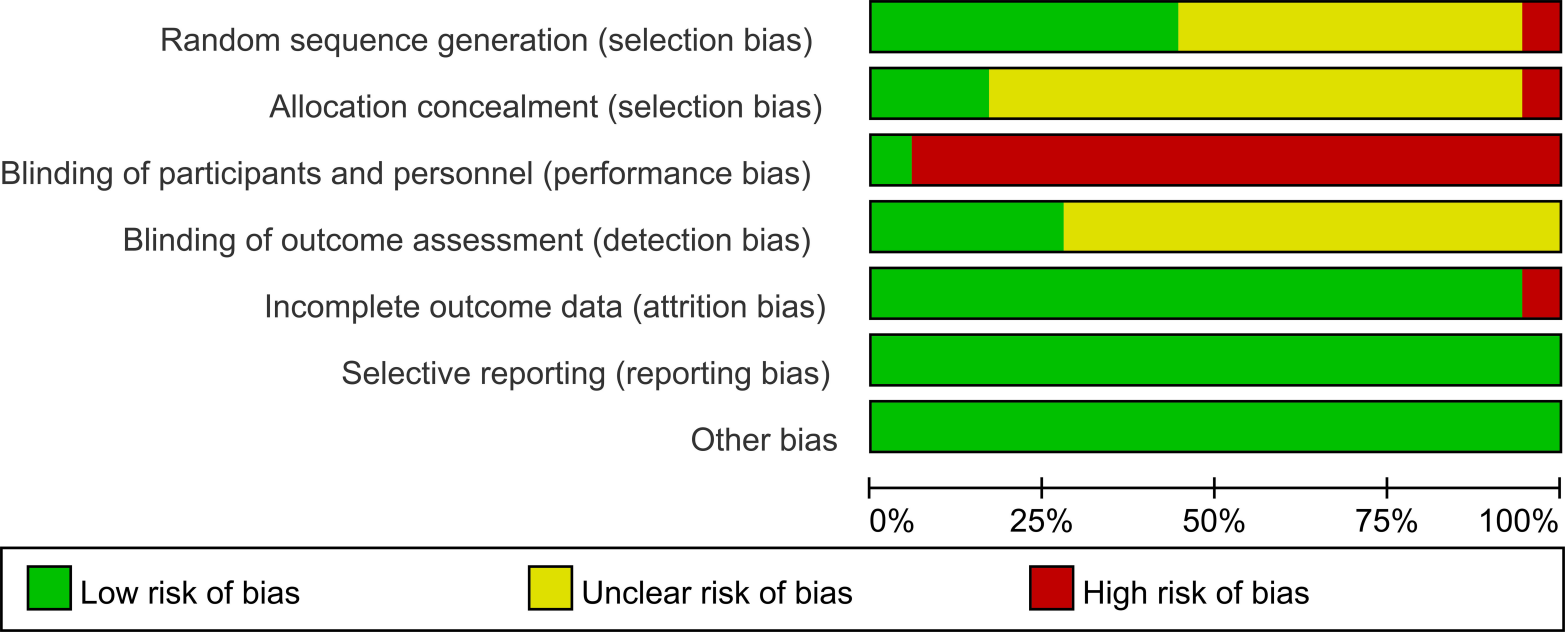
Risk of bias graph: review authors’ judgements about each risk of bias item presented as percentages across all included studies.

PEDro scores ranged from 4 to 9 (two excellent, eleven high, and five moderate) (**Figures 2**, **3**), while RoB 1.0 identified eight Grade A and ten Grade B studies. No low-quality or Grade C studies were included (**Figures 4**, **5**). The trials generally demonstrated strong methodological rigor in random sequence generation, baseline comparability, and incomplete outcome data management. Notably, blinding of participants and personnel universally presented a high risk of bias—an inherent constraint of physical exercise interventions rather than a methodological flaw. Overall, the risk of bias was low to moderate, supporting the validity of the quantitative synthesis.

## 7 Statistical analysis

All statistical analyses were performed using Review Manager (RevMan, version 5.4) and Stata/SE (version 15.0). Effect sizes were pooled as standardized mean differences (SMDs) with 95% confidence intervals (CIs) due to the variation in postural stability assessment tools. The magnitude of SMDs was classified as small (< 0.5), moderate (0.5–0.79), or large (≥ 0.8). Statistical heterogeneity was assessed via the *I*^2^ statistic, with values of < 25%, 25–50%, and > 50% representing low, moderate, and high heterogeneity, respectively.

A random-effects model was applied for all syntheses to account for anticipated clinical and methodological heterogeneity. Subgroup analyses explored potential sources of variance across predefined components. Additionally, a leave-one-out sensitivity analysis was conducted to evaluate the robustness of the pooled estimates. Potential publication bias was evaluated using Egger’s regression test, with statistical significance set at *p* < 0.05.

## 8 Assessment of publication bias

Egger’s regression test (Egger et al., 1997) indicated no significant publication bias for either dynamic [*t* = 1.36, *p* = 0.195, 95% CI (-0.942, 4.225)] or static postural stability outcomes [*t* = 1.33, *p* = 0.212, 95% CI (-1.386, 5.510)]. The regression intercepts for both domains firmly encompassed zero, confirming the absence of small-study effects and substantiating the robustness of the pooled estimates (**Table 3**).

**Table 3:** Results of Egger’s regression test for publication bias.

Outcome measure	Parameter	Coefficient	SE	*t*	*p*-value	95%CI
Dynamic postural stability	Slope	1.018	0.447	2.28	0.039	[0.059, 1.977]
	Intercept(bias)	-0.162	1.170	-0.14	0.892	[-2.670, 2.347]
Static postural stability	Slope	0.227	0.511	0.44	0.667	[-0.913, 1.367]
	Intercept(bias)	2.062	1.548	1.33	0.212	[-1.386, 5.510]

SE, standard error; CI, confidence interval. Note: The intercept (bias) represents the degree of asymmetry in the funnel plot. A *p*-value > 0.05 for the intercept indicates no significant evidence of publication bias.

## 9 Certainty of evidence assessment

The certainty of evidence for both dynamic and static postural stability was evaluated using the Grading of Recommendations Assessment, Development and Evaluation (GRADE) approach via GRADEpro GDT software (Guyatt et al., 2008). Evidence derived from the included RCTs was downgraded based on five standard domains: risk of bias, inconsistency, indirectness, imprecision, and publication bias. A GRADE evidence profile was subsequently generated to summarize the certainty of evidence alongside the absolute and relative effect estimates for each outcome (**Table 4**).

**Table 4:** Grade evidence profile for postural stability outcomes.

Certainty assessment											Certainty	Importance
Number of studies	Study design	Risk of bias	Inconsistency	Indirectness	Imprecision	Other considerations	Number of patients [NMT]	Number of patients [Control]	Relative Effect	Absolute Effect (95% CI)		
Dynamic posture stability												
15	randomized trials	serious^a,b^	not serious^c^	not serious^d^	not serious^e^	strong association	261	251	–	SMD **0.96 higher** (0.7 higher to 1.22 higher)	⊕⊕⊕⊕ High^a,b,c,d,e^	Critical
Static postural stability												
12	randomized trials	serious^a,f^	serious^g^	not serious^h^	not serious^i^	strong association	255	252	–	SMD **0.96 higher** (0.6 higher to 1.32 higher)	⊕⊕⊕◯ Moderate^a,f,g,h,i^	Critical

Explanations: ^a^Downgraded one level for risk of bias: Inherent lack of participant and personnel blinding in exercise interventions. ^b^Downgraded one level for risk of bias: Unclear allocation concealment in several trials. ^c^Not downgraded for inconsistency: Moderate heterogeneity (*I*^2^ = 44%) with consistent effect directions. ^d^Not downgraded for indirectness: Direct comparisons available. ^e^Not downgraded for imprecision: Optimal information size met (*N* = 516) with narrow 95% CIs. ^f^Downgraded one level for risk of bias: Unclear blinding of outcome assessors for subjective metrics (e.g., BESS). ^g^Downgraded one level for inconsistency: High statistical heterogeneity (*I*^2^ = 69%). ^h^Not downgraded for indirectness: Direct comparisons available. ^i^Not downgraded for imprecision: Optimal information size met (*N* = 507) with narrow 95% CIs. Bold values indicate the pooled effect size (Standardized Mean Difference) for the respective outcomes.

## 10 Results

### 10.1 Characteristics of control interventions

Across the 18 included trials, control groups predominantly underwent routine sport-specific training or standard warm-up protocols. Four studies utilized active controls: traditional resistance training [Gong et al. (2024)]; [Lmai et al (Imai et al., 2014)], kettlebell training like [Kim et al. (2024)], and an upper-extremity placebo protocol to mitigate the Hawthorne effect [Mitrousis et al. (2023)].

Notably, several control regimens inherently included neuromuscular-stimulating elements. For example, the aforementioned resistance and kettlebell protocols, as well as specific routine warm-ups involving multi-directional jumps [Pinzón-Romero et al. (2019)] or repetitive ankle hops [Shi et al. (2023)], possess plyometric or stability-enhancing characteristics. In this review, these overlapping elements are conceptualized as foundational “baseline exposures” typical of sports for young athletes conditioning, rather than methodological contamination.

### 10.2 Effects of NMT on dynamic postural stability

Fifteen articles (16 independent trials) comprising 516 participants (NMT: *n* = 265; Control: *n* = 251) evaluated dynamic postural stability. Assessments were primarily conducted using the Star Excursion Balance Test (SEBT), Y-Balance Test (YBT), or instrumented platforms.

**Figure 6:**
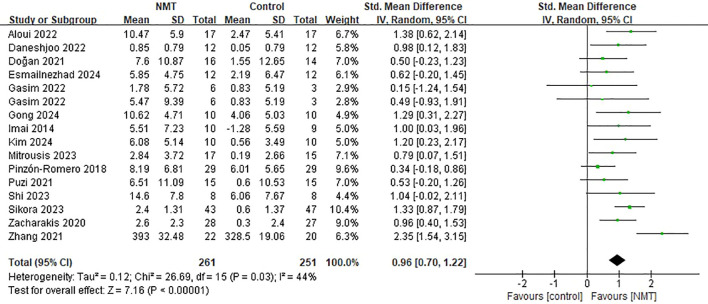
Forest plot of the effects of neuromuscular training (NMT) on dynamic postural stability in young athletes.

The pooled analysis demonstrated that NMT yielded a large and statistically significant improvement in dynamic postural stability compared to control conditions [SMD = 0.96, 95% CI (0.70, 1.22), *p* < 0.00001]. Moderate statistical heterogeneity was observed across the included trials (*I*^2^ = 44%, *p* = 0.03) (**Figure 6**).

#### 10.2.1 Subgroup analyses for dynamic postural stability

Subgroup analyses revealed that NMT consistently improved dynamic postural stability across all predefined components, with no significant subgroup differences detected for intervention modality (*p* = 0.55), duration (*p* = 0.24), outcome measures (*p* = 0.90), or participant age (*p* = 0.53) (**Table 5**).

**Table 5:** Subgroup analysis of NMT effects on dynamic postural stability.

Subgroup	Category	No. of studies	Participants(*N*)	SMD (95%CI)	*p*-value (Effect)	Heterogeneity (*I*^2^, *p*-value)
Intervention modality	Core stability Balance & proprioception Plyometrics Core stability + Balance & proprioception Balance & proprioception + Plyometrics Test for subgroup differences	4 4 4 3 1	78 235 79 90 30	0.76 [0.29, 1.23] 0.87 [0.42, 1.32] 1.16 [0.67, 1.65] 1.32 [0.27, 2.37] 0.53 [-0.20, 1.26] *χ*^2^ = 3.03, *p* = 0.55	0.002 0.0002< 0.00001 0.01 NA	0%, 0.46 62%, 0.05 0%, 0.75 79%, 0.008 NA *I*^2^ = 0%
Intervention duration	≤ 8 weeks > 8 weeks Test for subgroup differences	10 6	267 245	0.82 [0.57, 1.08] 1.21 [0.63, 1.79] *χ*^2^ = 1.40, *p* = 0.24	< 0.00001< 0.0001	0%, 0.77 73%, 0.002 *I*^2^ = 28.4%
Outcome measures	Active excursion-based tests Instrumented platforms Test for subgroup differences	12 4	385 127	0.97 [0.61, 1.32] 0.93 [0.56, 1.30] *χ*^2^ = 0.02, *p* = 0.90	< 0.00001< 0.00001	58%, 0.006 0%, 0.98 *I*^2^ = 0%
Participant age(years)	≤ 15 > 15 Test for subgroup differences	10 6	388 124	0.88 [0.63, 1.13] 1.11 [0.44, 1.78] *χ*^2^ = 0.40, *p* = 0.53	< 0.00001 0.001	23%, 0.23 61%, 0.02 *I*^2^ = 0%

SMD, standardized mean difference; CI, confidence interval; NA, not applicable. Note: The *p*-value (Effect) denotes the statistical significance of the pooled effect size within each subgroup. Heterogeneity within subgroups is reported using the *I*^2^ statistic and the corresponding Cochran’s *Q* test *p*-value. The test for subgroup differences assesses whether the effect sizes vary significantly across categories within a given component.

Regarding specific intervention modalities, significant improvements were noted across nearly all paradigms (SMDs ranging from 0.76 to 1.32). Only the combined balance/proprioception and plyometrics stratum failed to reach statistical significance [SMD = 0.53, 95% CI (-0.20, 1.26)], a finding likely attributable to the limited sample size within this stratum (n = 1). Furthermore, stability enhancements remained robust irrespective of whether interventions lasted ≤ 8 weeks (SMD = 0.82) or > 8 weeks (SMD = 1.21), whether assessed via active excursion or instrumented platforms, and regardless of the athletes being ≤ 15 or > 15 years of age.

#### 10.2.2 Sensitivity and heterogeneity analyses for dynamic postural stability

**Figure 7:**
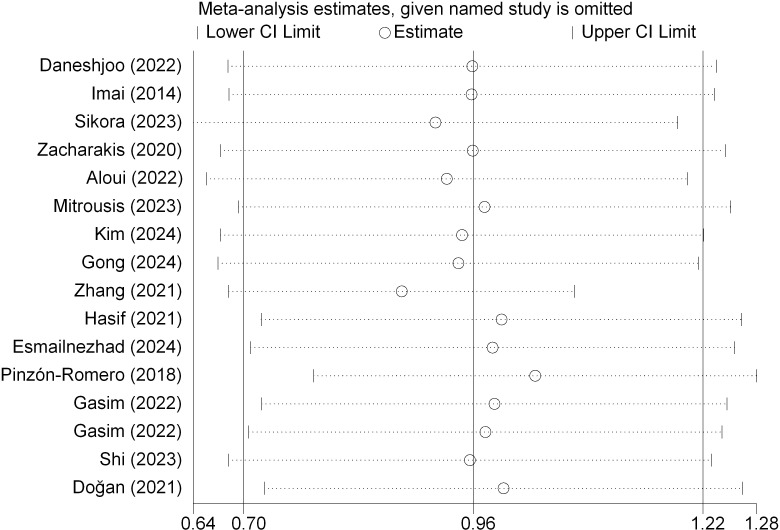
Leave-one-out sensitivity analysis for dynamic postural stability.

A leave-one-out sensitivity analysis confirmed the robustness of the primary findings; omitting any single trial did not alter the statistical significance of the pooled effect size (**Figure 7**). Additionally, excluding studies with active control groups yielded a comparable estimate [SMD = 0.93, 95% CI (0.60, 1.27)].

**Figure 8:**
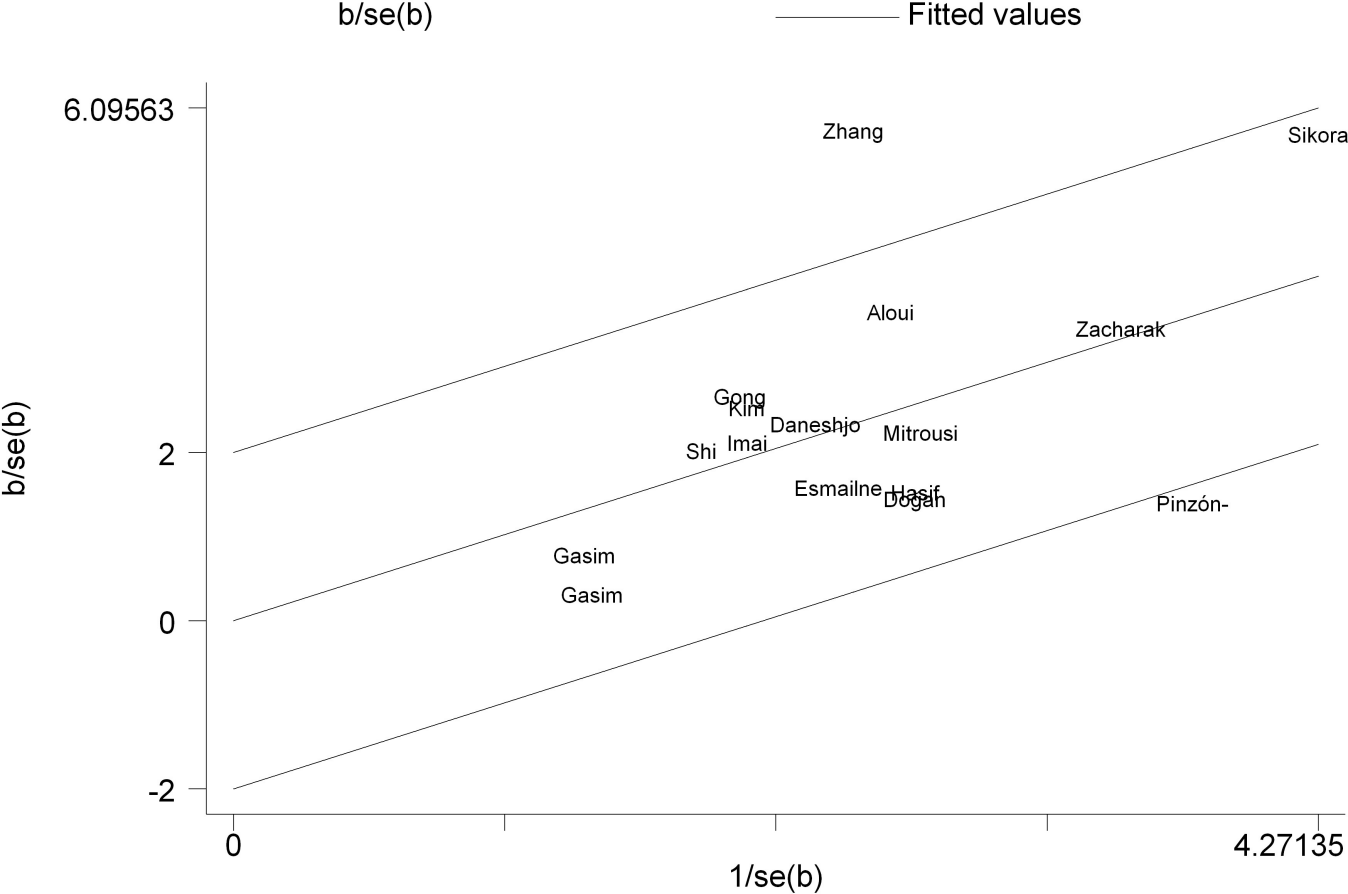
Galbraith plot for dynamic postural stability.

Galbraith plot analysis identified the trials by Zhang et al. (2021) and Pinzón-Romero et al. (2019) as the primary sources of the observed moderate heterogeneity (**Figure 8**). The hypothetical exclusion of these two trials eradicated statistical heterogeneity (*I*^2^ = 0%) without compromising the magnitude or significance of the overall effect [SMD = 0.96, 95% CI (0.76, 1.17)]. Consequently, all trials were retained in the final synthesis.

### 10.3 Effects of NMT on static postural stability

Twelve articles (12 independent trials) encompassing 507 participants (NMT: *n* = 255; Control: *n* = 252) evaluated static postural stability, utilizing tools such as the Balance Error Scoring System (BESS), single-leg stance tests, and stabilometric platforms.

**Figure 9:**
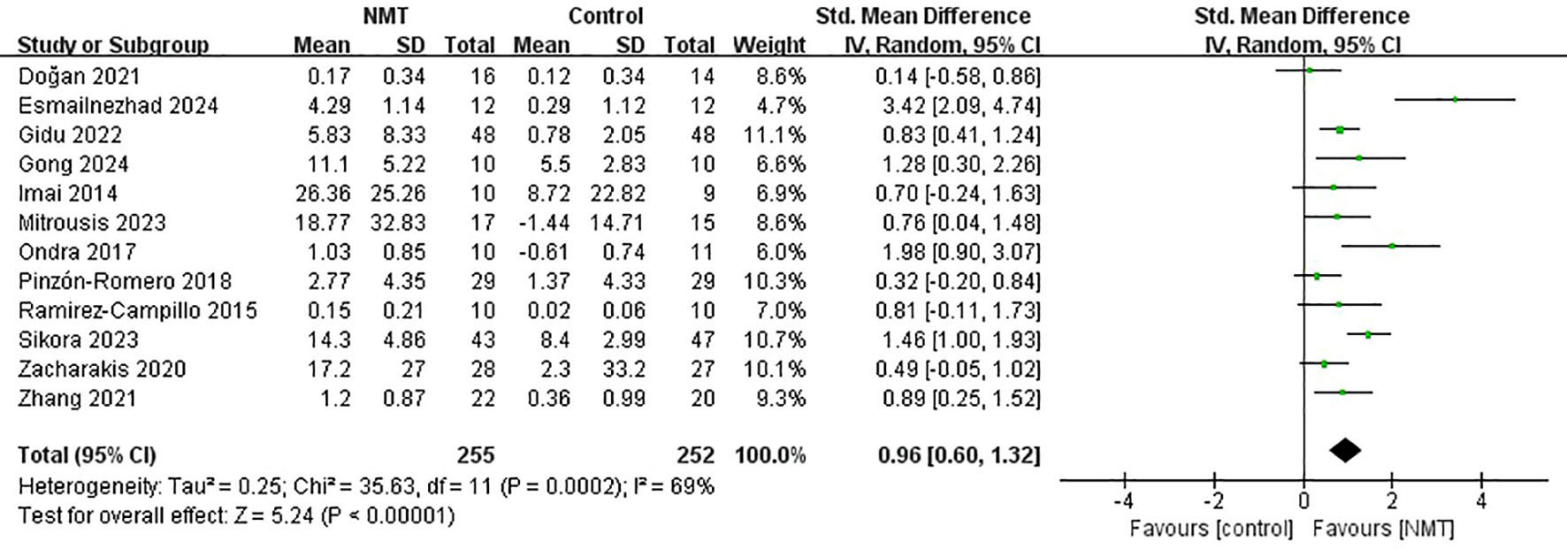
Forest plot of the effects of neuromuscular training (NMT) on static postural stability in young athletes.

The pooled analysis demonstrated a large and statistically significant enhancement in static postural stability following NMT relative to control regimens (SMD = 0.96, 95% CI [0.60, 1.32], *p* < 0.00001). Substantial statistical heterogeneity was observed across these trials (*I*^2^ = 69%, *p* = 0.0002) (**Figure 9**).

#### 10.3.1 Subgroup analyses for static postural stability

In contrast to dynamic stability, subgroup analyses identified participant age as a significant moderating variable for static postural stability (*p* = 0.04). Specifically, young athletes > 15 years derived significantly greater static stability gains [SMD = 1.78, 95% CI (0.79, 2.77)] compared to those ≤ 15 years [SMD = 0.71, 95% CI (0.39, 1.03)].

No significant subgroup differences were detected across intervention modalities (*p* = 0.70), duration (*p* = 0.68), or outcome measures (*p* = 0.52), all of which consistently demonstrated positive NMT effects (**Table 6**).

**Table 6:** Subgroup analysis of NMT effects on static postural stability.

Subgroup	Category	No. of Studies	Participants(*N*)	SMD(95%CI)	*p*-value (Effect)	Heterogeneity (*I*^2^, *p*-value)
Intervention modality	Core stability Balance & proprioception Plyometrics Core stability + Balance & proprioception Test for subgroup differences	3 6 1 2	69 352 20 66	0.64 [-0.02, 1.29] 0.90 [0.47, 1.32] 0.81 [-0.11, 1.73] 2.08 [-0.39, 4.56] *χ*^2^ = 1.40, *p* = 0.70	0.06< 0.0001 NA 0.10	42%, 0.18 70%, 0.005 NA 91%, 0.0007 *I*^2^ = 0%
Intervention duration	≤ 8 weeks > 8 weeks Test for subgroup differences	6 6	257 250	0.89 [0.33, 1.45] 1.05 [0.56, 1.54] *χ*^2^ = 0.17, *p* = 0.68	0.002< 0.0001	74%, 0.002 65%, 0.0001 *I*^2^ = 0%
Outcome measures	Instrumented platforms Clinical scoring scales Timed balance tests Test for subgroup differences	5 4 3	180 220 107	1.00 [0.37, 1.63] 1.13 [0.35, 1.90] 0.70 [0.30, 1.09] *χ*^2^ = 1.30, *p* = 0.52	0.002 0.004 0.0006	61%, 0.01 84%, 0.0004 0%, 0.38 *I*^2^ = 0%
Participant age(years)	≤ 15 > 15 Test for subgroup differences	8 4	400 107	0.71 [0.39, 1.03] 1.11 [0.44, 1.78] *χ*^2^ = 4.06, *p* = 0.04	< 0.0001 0.0004	55%, 0.03 76%, 0.006 *I*^2^ = 75.4%

SMD, standardized mean difference; CI, confidence interval; NA, not applicable. Note: The *p*-value (Effect) denotes the statistical significance of the pooled effect size within each subgroup. Heterogeneity within subgroups is reported using the *I*^2^ statistic and the corresponding Cochran’s *Q* test *p*-value. The test for subgroup differences assesses whether the effect sizes vary significantly across categories within a given component. Highlighted by the age subgroup analysis, participant age was identified as a statistically significant moderator (*p* = 0.04).

#### 10.3.2 Meta-regression analysis for static postural stability

To further elucidate the substantial heterogeneity observed in static postural stability (*I*^2^ = 69%), univariate meta-regression analyses were performed. Based on a component-based coding strategy, primary training components (core stability, balance and proprioception, and plyometrics) were entered as independent dichotomous covariates. The results demonstrated that none of the specific NMT modalities significantly predicted the magnitude of static stability improvements (all *p* > 0.05). Furthermore, these covariates accounted for no additional between-study variance (Adjusted *R*^2^ = 0%), suggesting that the specific selection of primary training modality is not a significant source of the observed statistical heterogeneity (**Table 7**).

**Table 7:** Univariate meta-regression analysis of primary NMT components on static postural stability.

Covariate (Primary component)	Coefficient	SE	95% CI	*t*-value	*p*-value	Adjusted *R*^2^ (%)
Core stability	0.21	0.48	[-0.87, 1.28]	0.43	0.679	0
Balance & proprioception	0.40	0.49	[-0.70, 1.49]	0.80	0.440	0
Plyometrics	-0.19	0.90	[-2.18, 1.81]	-0.21	0.838	0

SE, standard error; CI, confidence interval. Note: Each covariate was coded as a dichotomous variable (1 = presence of the component, 0 = absence of the component) and analyzed in a separate univariate restricted maximum likelihood (REML) model. Negative Adjusted *R*^2^ values obtained from the Stata output were reported as 0%, indicating no proportion of the between-study variance (*I*^2^ = 69%) was explained by the covariate.

#### 10.3.3 Sensitivity and heterogeneity analyses for static postural stability

**Figure 10:**
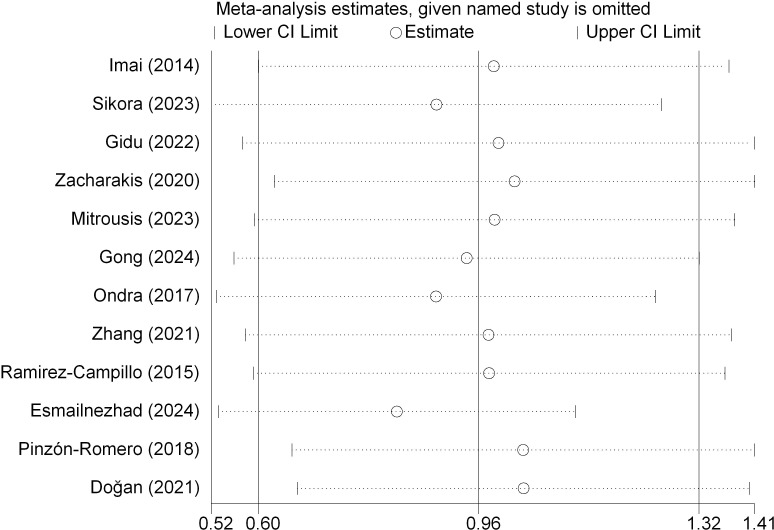
Leave-one-out sensitivity analysis for static postural stability.

A leave-one-out sensitivity analysis confirmed the robustness of the pooled estimate; the overall effect remained significant regardless of the omission of any single trial (**Figure 10**). Furthermore, excluding studies with active control interventions yielded a comparable and robust effect size [SMD = 1.00, 95% CI (0.55, 1.45)].

**Figure 11:**
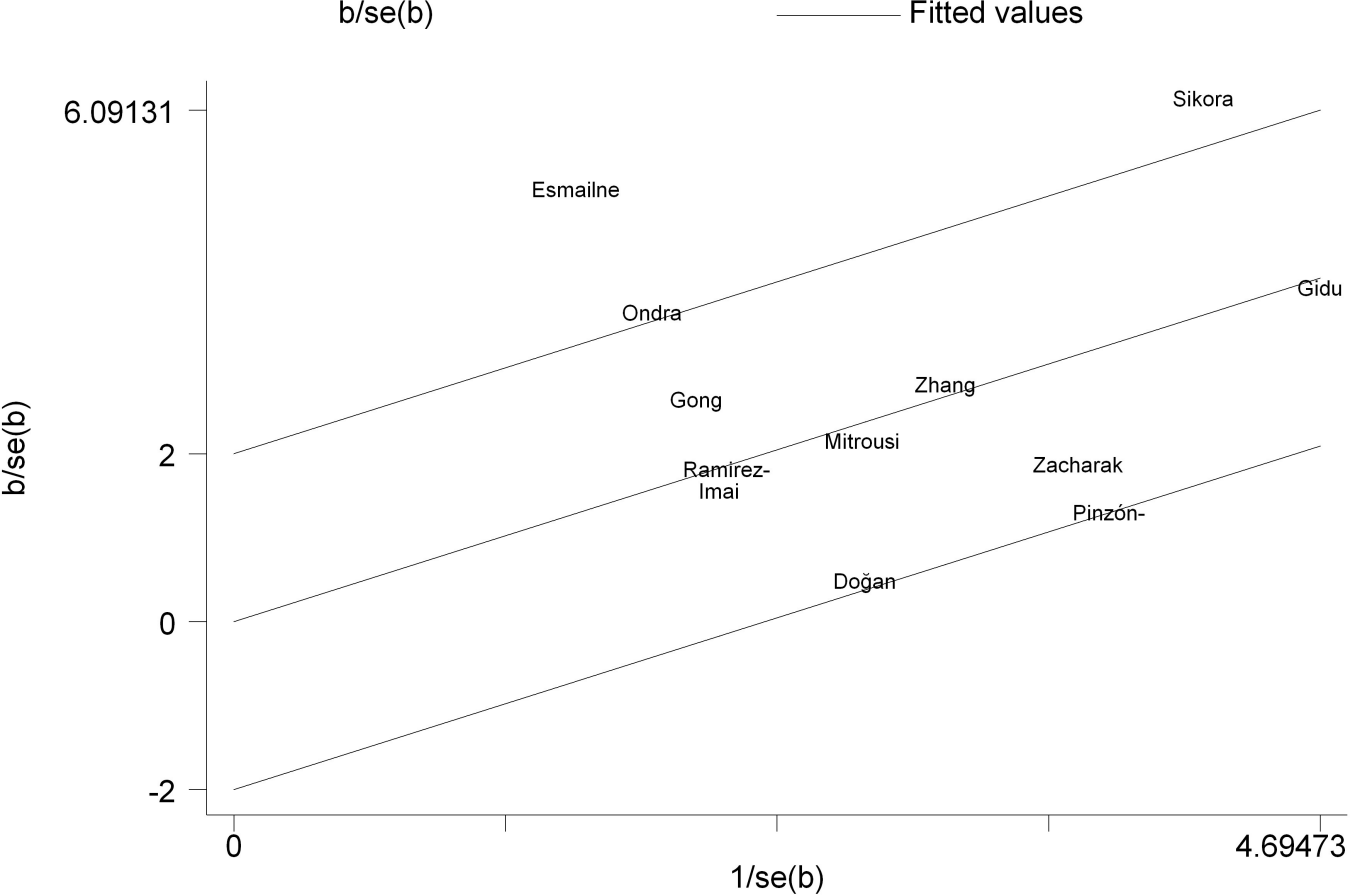
Galbraith plot for static postural stability.

Galbraith plot analysis identified the trials by Esmailnezhad et al. (2024) and Sikora et al (Sikora and Linek, 2022). as the principal sources of the high heterogeneity. Additionally, the studies by Ondra et al. (2017), Doğan et al (Dogan and Savaş, 2021), and Pinzón-Romero et al. (2019) were positioned on the 95% confidence boundaries, indicating them as potential secondary sources of variance (**Figure 11**). The hypothetical exclusion of these two outliers substantially reduced statistical heterogeneity to an acceptable level (*I*^2^ = 28%) while maintaining a significant pooled effect [SMD = 0.71, 95% CI (0.46, 0.97)]. Consistent with the dynamic stability approach, all trials were retained in the final quantitative synthesis.

## 11 Discussion

This meta-analysis demonstrates that neuromuscular training (NMT) effectively enhances both dynamic and static postural stability in young athletes, yielding large effect sizes (SMD = 0.96 for both). These findings corroborate previous reviews by Williams et al. (2021) and Gebel et al. (2018), reinforcing NMT as a foundational strategy for optimizing body control in sports for young athletes. However, beyond confirming overall efficacy, our synthesis elucidates several critical nuances previously overlooked in the literature.

While prior research frequently conflated dynamic and static stability (Williams et al., 2021), our distinct analyses reveal that these metrics are governed by different moderating variables. Notably, participant age emerged as a significant moderator for static stability adaptations. Unlike previous analyses that provided generalized conclusions regarding motor control, identifying this age-dependent response (>15 vs. ≤15 years) reconciles conflicting outcomes in individual trials. This differentiation underscores the decisive role of vestibular and neurophysiological maturation in dictating specific training adaptations (Viel et al., 2009; Myer et al., 2013).

Furthermore, this study highlights the comparable efficacy across diverse NMT modalities. While previous reviews primarily evaluated the dose-response of isolated balance training (Gebel et al., 2018), modern athletic practice demands integrative approaches where stability is coupled with core control and explosive power. By demonstrating that core stability, plyometrics, and combined regimens yield similar stability gains, our findings support the flexible implementation of NMT. This empowers strength and conditioning practitioners to tailor interventions based on sport-specific demands and logistical constraints, rather than adhering to rigid, single-modality protocols.

### 11.1 Mechanisms and modulators of dynamic postural stability

The substantial enhancement in dynamic postural stability (SMD = 0.96) is likely attributable to NMT’s capacity to optimize feedforward and feedback neuromuscular regulation (Wang et al., 2024). By systematically integrating plyometrics, core stability, and proprioceptive exercises, NMT increases muscular pre-activation and dynamic lower-extremity joint stiffness. These neurologically driven adaptations are critical for counteracting high-velocity kinetic perturbations during athletic maneuvers such as cutting and landing (Wikstrom et al., 2005; Pau et al., 2019). Importantly, this effect size (SMD = 0.96) transcends mere statistical significance, representing a clinical threshold capable of mitigating injury risk. In biomechanical terms, an improvement of nearly one standard deviation is sufficient to rectify postural deficits that are prospectively linked to increased ACL strain and ankle inversion trauma (Hewett et al., 2005; Plisky et al., 2006; Emery et al., 2015).

Subgroup analyses revealed a potential dose-response trend, with interventions exceeding 8 weeks yielding substantially larger effects than shorter protocols (SMD = 1.21 vs. 0.82). While initial stability gains (< 8 weeks) are typically driven by enhanced synaptic transmission and motor unit synchronization, prolonged training is necessary to facilitate deeper neuroplasticity and structural tendon adaptations (Fort-Vanmeerhaeghe et al., 2016; Hammami et al., 2025). Regarding intervention typologies, combining core stability with balance/proprioception training produced the largest effect (SMD = 1.32), reinforcing the clinical paradigm that proximal core stability facilitates distal mobility (Akuthota and Nadler, 2004; Behm et al., 2010). Plyometric training also elicited robust adaptations (SMD = 1.16) by overloading the stretch-shortening cycle (SSC) (Makaruk and Sacewicz, 2010). Importantly, the lack of significant variance across distinct modalities indicates that practitioners can safely tailor NMT prescriptions based on contextual constraints without compromising efficacy.

The observed moderate heterogeneity (*I*^2^ = 44%) was largely attributable to baseline physiological variations among the athletic cohorts. The pronounced effect size in Zhang et al. (2021) suggests a “superimposed effect” in elite dancers, where highly specialized NMT optimized neural pruning and motor automaticity beyond already exceptional baseline capacities (Issurin, 2010; Dayan and Cohen, 2011). Conversely, the attenuated effect observed by Pinzón-Romero et al. (2019) likely reflects a “physiological ceiling” inherent to competitive roller skaters (Hrysomallis, 2011). This dilution was further compounded by the control group’s active participation in multi-directional jumping, highlighting the methodological complexities of isolating NMT effects against the baseline exposures of sports conditioning for young athletes (Wachholz et al., 2020).

### 11.2 Mechanisms and modulators of static postural stability

The substantial improvement in static postural stability following NMT (SMD = 0.96) underscores its efficacy in optimizing the central nervous system’s sensory integration. Unlike dynamic stability, which relies heavily on feedforward motor execution and reflex stiffness, static postural control is fundamentally governed by the continuous integration and reweighting of somatosensory, visual, and vestibular inputs (Hof, 2008). NMT enhances the acuity of muscle spindles and articular mechanoreceptors, thereby facilitating a more efficient “sensory reweighting” process that shifts reliance from visual dominance to precise proprioceptive feedback during static stances (Peterka, 2002). Similar to the findings for dynamic stability, the large effect size observed in static postural control (SMD = 0.96) holds substantial clinical relevance. From a functional perspective, a nearly one-standard-deviation improvement in static sway metrics is associated with enhanced joint position sense and a reduced incidence of lateral ankle sprains, particularly in sports requiring prolonged unipedal stability (Tropp et al., 1984; McGuine et al., 2000; Hrysomallis, 2011).

Crucially, our subgroup analysis identified participant age as a significant moderator for static stability adaptations, with older youth (> 15 years) exhibiting an exceptionally large effect (SMD = 1.78) compared to their younger counterparts (SMD = 0.71). This age-dependent disparity aligns with the ontogeny of human postural control. The maturation of vestibular networks and advanced somatosensory integration pathways typically reaches adult-like proficiency during mid-adolescence (approximately 15–16 years) (Viel et al., 2009). Younger athletes inherently exhibit greater visual dependency for postural orientation; consequently, they may lack the requisite neural maturity to fully exploit the proprioceptive enhancements induced by NMT (Polastri and Barela, 2013). In contrast, post-pubertal athletes possess the mature neural architecture necessary to efficiently process and translate NMT-induced proprioceptive gains into superior static postural control (Myer et al., 2013; Gebel et al., 2020).

Beyond the maturation-dependent differences identified in our subgroup analysis, we further employed univariate meta-regression to verify whether the diverse NMT architectures themselves contributed to the observed high heterogeneity (*I*^2^ = 69%) in static stability. The results demonstrated that the inclusion of specific training components—core stability (*p* = 0.679), balance and proprioception (*p* = 0.440), and plyometrics (*p* = 0.838)—did not significantly moderate the intervention efficacy. Notably, the *R*^2^ value of 0% for all analyzed modalities systematically rules out intervention typology as a primary driver of the outcome dispersion. These regression findings reinforce the hypothesis that the “neuromuscular lag” and subsequent recovery are inherently tied to the ontogeny of sensory integration pathways, rather than the specific NMT modalities employed (Viel et al., 2009; McKay et al., 2016; Faude et al., 2017). Consequently, practitioners are afforded significant flexibility to tailor NMT prescriptions to specific athletic contexts without compromising the magnitude of static stability gains.

The substantial heterogeneity observed in this domain (*I*^2^ = 69%) was primarily driven by extreme sport-specific baselines and methodological sensitivities. The pronounced effect in Esmailnezhad et al. (2024) likely reflects the unique neuromuscular demands of wrestling in young athletes, where NMT synergized with grappling-specific tactile and proprioceptive conditioning to produce an outsized adaptation (Chaabene et al., 2017). Conversely, the variance introduced by Sikora et al (Sikora and Linek, 2022). highlights the sensitivity of the assessment tools utilized; high-frequency stabilometric platforms capture micro-fluctuations in the center of pressure (COP) that remain undetected by subjective visual scoring systems (e.g., BESS), thereby amplifying data dispersion across the pooled analysis (Bell et al., 2011). Additionally, secondary variance emerged from trials situated on the Galbraith plot boundaries (Ondra et al. (2017); Doğan et al (Dogan and Savaş, 2021); Pinzón-Romero et al. (2019)), reflecting the compounding influences of atypical intervention durations, metric mismatches, and pre-existing physiological ceilings.

## 12 Limitations and methodological considerations

Several limitations warrant consideration. Primarily, the clinical heterogeneity of the control groups introduced potential co-intervention bias. Several trials utilized active controls (e.g., resistance or kettlebell regimens) or routine warm-ups containing plyometric elements (e.g., multi-directional jumps), which inherently stimulate neuromuscular pathways. Although this “dilution effect” likely attenuated the comparative effect sizes, our sensitivity analyses confirming robust stability gains upon excluding these trials reinforce the validity of the core findings. Furthermore, the moderate-to-high statistical heterogeneity observed (*I*^2^ = 44%-69%) reflects inherent discrepancies in sport-specific baselines (e.g., elite dancers vs. amateur athletes) and the diverse sensitivities of the assessment modalities employed (e.g., subjective BESS versus instrumented stabilometry).

Methodologically, the unfeasibility of blinding participants and personnel—an inherent constraint of physical exercise interventions—uniformly introduced performance bias across the included RCTs. Finally, the current evidence base is predominantly confined to short-term adaptations (typically < 12 weeks). There remains a critical paucity of longitudinal data delineating the long-term retention of NMT-induced stability enhancements and their interaction with the continuous neurophysiological maturation of young athletes.

## 13 Conclusion and practical recommendations

This meta-analysis confirms that neuromuscular training (NMT) effectively enhances both dynamic and static postural stability in young athletes. These adaptations are critical for optimizing motor control and mitigating lower-extremity injury risks during athletic development. Based on the quantitative synthesis and mechanistic insights, we propose the following evidence-based recommendations for clinical and athletic practitioners:

Age-Stratified Prescription: Tailor interventions to maturational windows. For athletes ≤ 15 years, prioritize foundational core and proprioceptive training to counteract adolescent motor incoordination. For those > 15 years, leverage their mature sensory integration by introducing high-intensity vestibular perturbations and visual occlusions.

Dose-Response Optimization and Maintenance: While an initial 8–12 weeks NMT cycle is required to consolidate neural adaptations, a season-long maintenance framework is critical to prevent detraining. Practitioners should integrate reduced-volume NMT (e.g., 1–2 sessions/week, or 10-minute micro-doses during routine warm-ups) throughout the competitive season to sustain optimal stability. Integrative Modalities: Adopt multi-component NMT architectures. Synergizing plyometrics, core stability, and balance training provides a more comprehensive stimulus to the central nervous system than isolated modalities.

Sport-Specific Contextualization: Integrate NMT with sport-specific technical demands. Executing stability tasks within specific athletic contexts (e.g., wearing skates or maintaining grappling stances) maximizes the ecological validity of the intervention and facilitates the direct transfer of stability gains to competitive performance.

## Data Availability

The original contributions presented in the study are included in the article/**Supplementary Material**. Further inquiries can be directed to the corresponding author.

## References

[B57] Akuthota V. Nadler S. F. (2004). Core strengthening. Arch. Phys. Med. Rehabil. 85, 86–92. doi: 10.1016/j.pmrj.2011.06.001. PMID: 15034861

[B37] Aloui G. Hermassi S. Bartels T. Hayes L. D. Bouhafs E. G. Chelly M. S. . (2022). Combined plyometric and short sprint training in u-15 male soccer players: effects on measures of jump, speed, change of direction, repeated sprint, and balance. Front. Physiol. 13, 757663. doi: 10.3389/fphys.2022.757663. PMID: 35250606 PMC8895237

[B56] Behm D. G. Drinkwater E. J. Willardson J. M. Cowley P. M. (2010). The use of instability to train the core musculature. Appl. Physiol. Nutr. Metab. 35, 91–108. doi: 10.1139/h09-127. PMID: 20130672

[B22] Behm D. G. Muehlbauer T. Kibele A. Granacher U. (2015). Effects of strength training using unstable surfaces on strength, power and balance performance across the lifespan: a systematic review and meta-analysis. Sports Med. 45, 1645–1669. doi: 10.1007/s40279-015-0384-x. PMID: 26359066 PMC4656700

[B69] Bell D. R. Guskiewicz K. M. Clark M. A. Padua D. A. (2011). Systematic review of the balance error scoring system. Sports Health 3, 287–295. doi: 10.1177/1941738111403122. PMID: 23016020 PMC3445164

[B9] Borato L. A. Whatman C. Walters S. Read P. (2025). What do we know (and not know) about adolescent awkwardness in youth sports? A narrative review. Int. J. Sports Sci. Coaching 20, 2257–2267. doi: 10.1177/17479541251364101

[B68] Chaabene H. Negra Y. Bouguezzi R. Mkaouer B. Franchini E. Julio U. . (2017). Physical and physiological attributes of wrestlers: an update. J. Strength Conditioning Res. 31, 1411–1442. doi: 10.1519/jsc.0000000000001738. PMID: 28030533

[B29] Chandler J. Cumpston M. Li T. Page M. J. Welch V. (2019). Cochrane handbook for systematic reviews of interventions Vol. 4 (Hoboken: Wiley), 14651858.

[B24] Daneshjoo A. Hoseinpour A. Sadeghi H. Kalantari A. Behm D. G. (2022). The effect of a handball warm-up program on dynamic balance among elite adolescent handball players. Sports 10, 18. doi: 10.3390/sports10020018. PMID: 35202058 PMC8876563

[B60] Dayan E. Cohen L. G. (2011). Neuroplasticity subserving motor skill learning. Neuron 72, 443–454. doi: 10.1016/j.neuron.2011.10.008. PMID: 22078504 PMC3217208

[B39] Dogan O. Savaş S. (2021). Effect of an 8-weeks core training program applied to 12–14 years old basketball players on strength, balance and basketball skill. Pakistan J. Med. Health Sci. 15, 182–185.

[B46] Egger M. Smith G. D. Schneider M. Minder C. (1997). Bias in meta-analysis detected by a simple, graphical test. bmj 315, 629–634. doi: 10.1136/bmj.315.7109.629. PMID: 9310563 PMC2127453

[B20] Emery C. A. Roy T.-O. Whittaker J. L. Nettel-Aguirre A. Van Mechelen W. (2015). Neuromuscular training injury prevention strategies in youth sport: a systematic review and meta-analysis. Br. J. Sports Med. 49, 865–870. doi: 10.1136/bjsports-2015-094639. PMID: 26084526

[B31] Esmailnezhad S. Daneshmandi H. Samami N. Mirzaei B. (2024). The effect of Wrestling+ warm-up program on balance and proprioception of adolescent wrestlers. J. Kinesiology Exercise Sci. 34, 45–52. doi: 10.5604/01.3001.0054.6754. PMID: 42089262

[B14] Faigenbaum A. D. Farrell A. Fabiano M. Radler T. Naclerio F. Ratamess N. A. . (2011). Effects of integrative neuromuscular training on fitness performance in children. Pediatr. Exercise Sci. 23, 573–584. doi: 10.1123/pes.23.4.573. PMID: 22109781

[B17] Faude O. Rössler R. Petushek E. J. Roth R. Zahner L. Donath L. (2017). Neuromuscular adaptations to multimodal injury prevention programs in youth sports: a systematic review with meta-analysis of randomized controlled trials. Front. Physiol. 8, 791. doi: 10.3389/fphys.2017.00791. PMID: 29075200 PMC5643472

[B55] Fort-Vanmeerhaeghe A. Romero-Rodriguez D. Montalvo A. M. Kiefer A. W. Lloyd R. S. Myer G. D. (2016). Integrative neuromuscular training and injury prevention in youth athletes. Part I: Identifying risk factors. Strength Conditioning J. 38, 36–48. doi: 10.1519/ssc.0000000000000229. PMID: 38604988

[B25] Gasim Z. K. Cengizel E. Günay M. (2022). Core vs plyometric training effects on dynamic balance in young male soccer players. Rev. Bras. Med. do Esporte 28, 326–330. doi: 10.1590/1517-8692202228042021_0048

[B21] Gebel A. Lesinski M. Behm D. G. Granacher U. (2018). Effects and dose–response relationship of balance training on balance performance in youth: a systematic review and meta-analysis. Sports Med. 48, 2067–2089. doi: 10.1007/s40279-018-0926-0. PMID: 29736728

[B67] Gebel A. Prieske O. Behm D. G. Granacher U. (2020). Effects of balance training on physical fitness in youth and young athletes: a narrative review. Strength Conditioning J. 42, 35–44. doi: 10.1519/ssc.0000000000000548. PMID: 38604988

[B38] Gidu D. V. Badau D. Stoica M. Aron A. Focan G. Monea D. . (2022). The effects of proprioceptive training on balance, strength, agility and dribbling in adolescent male soccer players. Int. J. Environ. Res. Public Health 19, 2028. doi: 10.3390/ijerph19042028. PMID: 35206215 PMC8871985

[B32] Gong J. Gao H. Sui J. Qi F. (2024). The effect of core stability training on the balance ability of young male basketball players. Front. Physiol. 14, 1305651. doi: 10.21203/rs.3.rs-3295879/v1. PMID: 38250660 PMC10796723

[B13] Gu J. Zhang R. Zhang Y. Shaharudin S. (2025). Neuromuscular training for preventing knee injuries in female team athletes: a meta-analysis. Ann. Med. 57, 2581891. doi: 10.1080/07853890.2025.2581891. PMID: 41175154 PMC12581765

[B47] Guyatt G. H. Oxman A. D. Vist G. E. Kunz R. Falck-Ytter Y. Alonso-Coello P. . (2008). GRADE: an emerging consensus on rating quality of evidence and strength of recommendations. bmj 336, 924–926. doi: 10.1136/bmj.39489.470347.ad. PMID: 18436948 PMC2335261

[B54] Hammami A. Mahmoudi A. Selmi W. Negra Y. Rebai H. Granacher U. . (2025). Effects of neuromuscular versus plyometric training on physical fitness and mental well-being in male pubertal soccer players. Sci. Rep. 15, 43393. doi: 10.1038/s41598-025-30142-x. PMID: 41361237 PMC12689855

[B11] Hewett T. E. Ford K. R. Hoogenboom B. J. Myer G. D. (2010). Understanding and preventing acl injuries: current biomechanical and epidemiologic considerations-update 2010. North. Am. J. Sports Phys. Therapy: NAJSPT 5, 234. PMC3096145 21655382

[B2] Hewett T. E. Myer G. D. Ford K. R. Heidt R. S. Colosimo A. J. McLean S. G. . (2005). Biomechanical measures of neuromuscular control and valgus loading of the knee predict anterior cruciate ligament injury risk in female athletes: a prospective study. Am. J. Sports Med. 33, 492–501. doi: 10.1177/0363546504269591. PMID: 15722287

[B45] Higgins J. P. Altman D. G. Gøtzsche P. C. Jüni P. Moher D. Oxman A. D. . (2011). The Cochrane Collaboration’s tool for assessing risk of bias in randomised trials. bmj 343, d5928. doi: 10.1136/bmj.d5928. PMID: 22008217 PMC3196245

[B30] Higgins J. P. Li T. Deeks J. J. (2019). “ Choosing effect measures and computing estimates of effect,” in Cochrane handbook for systematic reviews of interventions. (Hoboken, NJ: John Wiley & Sons), 143–176.

[B63] Hof A. L. (2008). The ‘extrapolated center of mass’ concept suggests a simple control of balance in walking. Hum. Mov. Sci. 27, 112–125. doi: 10.1016/j.humov.2007.08.003 17935808

[B1] Horak F. B. (2006). Postural orientation and equilibrium: what do we need to know about neural control of balance to prevent falls? Age Ageing 35, ii7–ii11. doi: 10.1093/ageing/afl077. PMID: 16926210

[B61] Hrysomallis C. (2011). Balance ability and athletic performance. Sports Med. 41, 221–232. doi: 10.2165/11538560-000000000-00000. PMID: 21395364

[B27] Imai A. Kaneoka K. Okubo Y. Shiraki H. (2014). Effects of two types of trunk exercises on balance and athletic performance in youth soccer players. Int. J. Sports Phys. Ther. 9, 47. 24567855 PMC3924608

[B59] Issurin V. B. (2010). New horizons for the methodology and physiology of training periodization. Sports Med. 40, 189–206. doi: 10.2165/11319770-000000000-00000. PMID: 20199119

[B33] Kim J. Jaber H. Yim J. (2024). Comparison of the effects of compound training, plyometric exercises, and kettlebell exercises on strength, power, dynamic balance, and pitched ball velocity in 30 male high school baseball pitchers aged 16–19 years. Med. Sci. Monitor: Int. Med. J. Exp. Clin. Res. 30, e944623-1. doi: 10.12659/msm.944623. PMID: 39118306 PMC11321950

[B44] Maher C. G. Sherrington C. Herbert R. D. Moseley A. M. Elkins M. (2003). Reliability of the PEDro scale for rating quality of randomized controlled trials. Phys. Ther. 83, 713–721. doi: 10.1093/ptj/83.8.713 12882612

[B58] Makaruk H. Sacewicz T. (2010). Effects of plyometric training on maximal power output and jumping ability. Hum. Movement 11, 17–22. doi: 10.2478/v10038-010-0007-1

[B4] Malina R. M. Bouchard C. Bar-Or O. (2004). Growth, maturation, and physical activity ( Champaign, IL: Human Kinetics).

[B64] McGuine T. A. Greene J. J. Best T. Leverson G. (2000). Balance as a predictor of ankle injuries in high school basketball players. Clin. J. Sport Med. 10, 239–244. doi: 10.1097/00042752-200010000-00003. PMID: 11086748

[B8] McKay D. Broderick C. Steinbeck K. (2016). The adolescent athlete: a developmental approach to injury risk. Pediatr. Exercise Sci. 28, 488–500. doi: 10.1123/pes.2016-0021. PMID: 27705538

[B34] Mitrousis I. Bourdas D. I. Kounalakis S. Bekris E. Mitrotasios M. Kostopoulos N. . (2023). The effect of a balance training program on the balance and technical skills of adolescent soccer players. J. Sports Sci. Med. 22, 645. doi: 10.52082/jssm.2023.645. PMID: 38045735 PMC10690516

[B16] Myer G. D. Faigenbaum A. D. Chu D. A. Falkel J. Ford K. R. Best T. M. . (2011). Integrative training for children and adolescents: techniques and practices for reducing sports-related injuries and enhancing athletic performance. Physician Sportsmedicine 39, 74–84. doi: 10.3810/psm.2011.02.1854. PMID: 21378489

[B49] Myer G. D. Sugimoto D. Thomas S. Hewett T. E. (2013). The influence of age on the effectiveness of neuromuscular training to reduce anterior cruciate ligament injury in female athletes: a meta-analysis. Am. J. Sports Med. 41, 203–215. doi: 10.1177/0363546512460637. PMID: 23048042 PMC4160039

[B42] Ondra L. Nátěsta P. Bizovská L. Kuboňová E. Svoboda Z. (2017). Effect of in-season neuromuscular and proprioceptive training on postural stability in male youth basketball players. Acta Gymnica 47, 144–149. doi: 10.5507/ag.2017.019

[B28] Page M. J. McKenzie J. E. Bossuyt P. M. Boutron I. Hoffmann T. C. Mulrow C. D. . (2021). The PRISMA 2020 statement: an updated guideline for reporting systematic reviews. bmj 372, n71. doi: 10.31222/osf.io/v7gm2. PMID: 33782057 PMC8005924

[B3] Paillard T. (2019). Relationship between sport expertise and postural skills. Front. Psychol. 10, 1428. doi: 10.3389/fpsyg.2019.01428. PMID: 31293483 PMC6603331

[B7] Parry G. N. Williams S. McKay C. D. Johnson D. J. Bergeron M. F. Cumming S. P. (2024). Associations between growth, maturation and injury in youth athletes engaged in elite pathways: a scoping review. Br. J. Sports Med. 58, 1001–1010. doi: 10.1136/bjsports-2024-108233. PMID: 39209526 PMC11420720

[B6] Parsons J. (2014). Assessing and modifying neuromuscular risk factors for anterior cruciate ligament injury in female athletes. (Winnipeg, MB, Canada: University of Manitoba).

[B52] Pau M. Porta M. Arippa F. Pilloni G. Sorrentino M. Carta M. . (2019). Dynamic postural stability, is associated with competitive level, in youth league soccer players. Phys. Ther. Sport 35, 36–41. doi: 10.1016/j.ptsp.2018.11.002. PMID: 30419410

[B18] Peterka R. J. (2002). Sensorimotor integration in human postural control. J. Neurophysiol. 88, 1097–1118. doi: 10.1152/jn.2002.88.3.1097. PMID: 12205132

[B26] Pinzón-Romero S. Vidarte-Claros J. A. Sánchez-Delgado J. C. (2019). Effects of a proprioceptive physical exercise program on balance in young skaters aged between 11 to 15 years. Arch. Med. Deporte 36, 166–171.

[B53] Plisky P. J. Rauh M. J. Kaminski T. W. Underwood F. B. (2006). Star excursion balance test as a predictor of lower extremity injury in high school basketball players. J. Orthopaedic Sports Phys. Ther. 36, 911–919. doi: 10.2519/jospt.2006.2244. PMID: 17193868

[B66] Polastri P. F. Barela J. A. (2013). Adaptive visual re-weighting in children’s postural control. PloS One 8, e82215. doi: 10.1371/journal.pone.0082215. PMID: 24324766 PMC3853149

[B40] Puzi M. H. B. M. Choo L. A. (2021). The effect of six weeks cobagi training on coordination, dynamic balance & agility of adolescent handball players. Pedagogy Phys. Culture Sports 25, 31–38. doi: 10.15561/26649837.2021.0105

[B5] Quatman C. E. Ford K. R. Myer G. D. Hewett T. E. (2006). Maturation leads to gender differences in landing force and vertical jump performance: a longitudinal study. Am. J. Sports Med. 34, 806–813. doi: 10.1177/0363546505281916. PMID: 16382009

[B43] Ramírez-Campillo R. Gallardo F. Henriquez-Olguín C. Meylan C. M. Martínez C. Álvarez C. . (2015). Effect of vertical, horizontal, and combined plyometric training on explosive, balance, and endurance performance of young soccer players. J. Strength Conditioning Res. 29, 1784–1795. doi: 10.1519/jsc.0000000000000827. PMID: 25559903

[B35] Shi Z. Xuan S. Deng Y. Zhang X. Chen L. Xu B. . (2023). The effect of rope jumping training on the dynamic balance ability and hitting stability among adolescent tennis players. Sci. Rep. 13, 4725. doi: 10.1038/s41598-023-31817-z. PMID: 36959249 PMC10036319

[B36] Sikora D. Linek P. (2022). Effect of a 10-week sensomotor exercise program on balance and agility in adolescent football players: a randomised control trial. Appl. Sci. 13, 89. doi: 10.3390/app13010089. PMID: 30654563

[B65] Tropp H. Ekstrand J. Gillquist J. (1984). Stabilometry in functional instability of the ankle and its value in predicting injury. Med. Sci. Sports Exercise 16, 64–66. doi: 10.1249/00005768-198401000-00013 6708781

[B19] Vacchini V. Brafa B. Nicotra R. Capelli E. Signorini S. Gasparroni V. . (2025). Improving neuroplasticity and quality of life in children with cerebral palsy: a customized intensive motor training protocol integrating the HABIT-ILE approach. Front. Rehabil. Sci. 6, 1613103. doi: 10.3389/fresc.2025.1613103. PMID: 41158535 PMC12554712

[B48] Viel S. Vaugoyeau M. Assaiante C. (2009). Adolescence: a transient period of proprioceptive neglect in sensory integration of postural control. Motor Control 13, 25–42. doi: 10.1123/mcj.13.1.25. PMID: 19246776

[B62] Wachholz F. Tiribello F. Mohr M. van Andel S. Federolf P. (2020). Adolescent awkwardness: Alterations in temporal control characteristics of posture with maturation and the relation to movement exploration. Brain Sci. 10, 216. doi: 10.3390/brainsci10040216. PMID: 32260555 PMC7226109

[B50] Wang P. Liu Y. Chen C. (2024). Effects of neuromuscular training on dynamic balance ability in athletes: a systematic review and meta-analysis. Heliyon 10, e35823. doi: 10.1016/j.heliyon.2024.e35823. PMID: 39220942 PMC11365420

[B51] Wikstrom E. A. Tillman M. D. Smith A. N. Borsa P. A. (2005). A new force-plate technology measure of dynamic postural stability: the dynamic postural stability index. J. Athletic Training 40, 305. doi: 10.4085/1062-6050-40.4.305 PMC1323292 16404452

[B10] Williams M. D. Ramirez-Campillo R. Chaabene H. Moran J. (2021). Neuromuscular training and motor control in youth athletes: a meta-analysis. Perceptual Motor Skills 128, 1975–1997. doi: 10.1177/00315125211029006. PMID: 34293993 PMC8414837

[B12] Wordeman S. C. (2014). Effects of Neuromuscular Training in Anterior Cruciate Ligament-Reconstructed Subjects (Columbus, OH: The Ohio State University).

[B15] Yang F. Lu C. Yun X. Qian C. (2025). Effects of neuromuscular training on stability in volleyball athletes: a systematic review and meta-analysis. Front. Sports Active Living 7, 1724934. doi: 10.3389/fspor.2025.1724934. PMID: 41602798 PMC12832796

[B41] Zacharakis E. D. Bourdas D. I. Kotsifa M. I. Bekris E. M. Velentza E. T. Kostopoulos N. I. (2020). Effect of balance and proprioceptive training on balancing and technical skills in 13-14-year-old youth basketball players. J. Phys. Educ. Sport 20, 2487–2500. doi: 10.7752/jpes.2020.05340

[B23] Zhang M. Ma H. Liu Z. Smith D. M. Wang X. (2021). The effects of a 10-week neuromuscular training on postural control in elite youth competitive ballroom dancers: a randomized controlled trial. Front. Physiol. 12, 636209. doi: 10.3389/fphys.2021.636209. PMID: 33841172 PMC8027106

