# Supplementary material for: Efficacy of neuromuscular training for enhancing postural stability in young athletes: a systematic review and meta-analysis
Source: Front Physiol. 2026 May 21;17:1827904. doi: 10.3389/fphys.2026.1827904 (PMC13233248; doi:10.3389/fphys.2026.1827904)
Supplement: Supplementary file 1 [file DataSheet1.docx]

**Appendix: Search Strategies for All Databases**

**Review Title:** Efficacy of Neuromuscular Training for Enhancing Postural Stability in Youth Athletes: A Systematic Review and Meta-Analysis

**Search Date:** Through November 1, 2025

**1. PubMed (via NCBI)**

**1 Population**

("Adolescent"[Mesh] OR "Athletes"[Mesh] OR "youth"[tiab] OR "adolescent*"[tiab] OR "teenager*"[tiab] OR "young athlete*"[tiab] OR "student athlete*"[tiab])

**2 Intervention**

("Neuromuscular training"[tiab] OR "integrative neuromuscular training"[tiab] OR "plyometric training"[tiab] OR "balance training"[tiab] OR "proprioceptive training"[tiab] OR "core stability training"[tiab])

**3 Outcome**

("Postural Balance"[Mesh] OR "postural stability"[tiab] OR "postural control"[tiab] OR "dynamic balance"[tiab] OR "static balance"[tiab] OR "Y-balance test"[tiab] OR "Star Excursion Balance Test"[tiab] OR "SEBT"[tiab] OR "BESS"[tiab] OR "stabilometry"[tiab])

**4 Design**

(randomized controlled trial[pt] OR controlled clinical trial[pt] OR randomized[tiab] OR placebo[tiab] OR "clinical trials as topic"[mesh:noexp] OR randomly[tiab] OR trial[ti]) NOT (animals[mesh] NOT humans[mesh])

**Final**

1 AND 2 AND 3 AND 4

**2. Embase (via Elsevier)**

Strategy: ('adolescent'/exp OR 'athlete'/exp OR 'youth':ti,ab OR 'adolescent*':ti,ab OR 'young athlete*':ti,ab) AND ('neuromuscular training'/exp OR 'plyometric training'/exp OR 'proprioceptive training'/exp OR 'balance training'/exp OR 'core stability training':ti,ab) AND ('postural stability'/exp OR 'body equilibrium'/exp OR 'y balance test':ti,ab OR 'star excursion balance test':ti,ab OR 'sebt':ti,ab OR 'bess':ti,ab OR 'stabilometry':ti,ab) AND ('randomized controlled trial'/exp OR 'randomization'/exp OR 'rct':ti,ab)

**3. Cochrane Library (Trials)**

1 [Mesh descriptor: [Adolescent] explode all trees]

2 [Mesh descriptor: [Athletes] explode all trees]

3 (youth OR adolescent* OR "young athlete*"):ti,ab,kw

4 1 OR 2 OR 3

5 ("neuromuscular training" OR "plyometric*" OR "balance training" OR "proprioceptive training" OR "core stability"):ti,ab,kw

6 [Mesh descriptor: [Postural Balance] explode all trees]

7 ("postural stability" OR "postural control" OR "Y-balance test" OR "SEBT" OR "BESS"):ti,ab,kw

8 6 OR 7

9 4 AND 5 AND 8

**4. Web of Science (Core Collection)**

TS= ((adolescent* OR youth OR "young athlete*" OR "student athlete*") AND ("neuromuscular training" OR "integrative neuromuscular training" OR "plyometric*" OR "balance training" OR "proprioceptive training" OR "core stability") AND ("postural stability" OR "postural control" OR "dynamic balance" OR "static balance" OR "Y-balance test" OR "SEBT" OR "BESS") AND ("randomized controlled trial" OR "RCT" OR "randomly"))

**5. Scopus (via Elsevier)**

TITLE-ABS-KEY ((adolescent* OR youth OR "young athlete*") AND ("neuromuscular training" OR "plyometric*" OR "balance training" OR "proprioceptive training") AND ("postural stability" OR "postural control" OR "Y-balance test" OR "SEBT") AND ("randomized controlled trial" OR "RCT"))
